# Delayed femoral artery injury caused by heterotopic ossification: a rare case report and review of the literature

**DOI:** 10.1186/s12891-024-07609-5

**Published:** 2024-06-20

**Authors:** Yan-Hui Li, Mingxi Liu, Chuanyang Zhou, Lei Tan

**Affiliations:** 1 Department of Cardiology and Echocardiography, the First Hospital of Jilin University, Changchun, 130021 China; 2 Department of Orthopedic Trauma, the First Hospital of Jilin University, No. 71 Xinmin Street, Changchun, Jilin 130021 China

**Keywords:** Artery injury, Organization of hematoma, Heterotopic ossification, Femoral shaft fracture

## Abstract

**Background:**

Arterial injury caused by heterotopic ossification (HO) following fractures is rarely reported, yet it can have catastrophic consequences. This case report presents a unique instance of femoral artery injury and hematoma organization, occurring a decade after intramedullary nail fixation for a femoral shaft fracture complicated by HO.

**Case presentation:**

A 56-year-old male presented with right femoral artery injury and organized hematoma, a decade after suffering bilateral femoral shaft fractures with mild head injury in a traffic accident. He had received intramedullary nailing for the right femoral shaft fracture and plate fixation for the left side in a local hospital. Physical examination revealed two firm, palpable masses with clear boundaries, limited mobility, and no tenderness. Peripheral arterial pulses were intact. Radiography demonstrated satisfactory fracture healing, while a continuous high-density shadow was evident along the inner and posterior aspect of the right thigh. Computed tomography angiography identified a large mixed-density mass (16.8 × 14.8 × 20.7 cm) on the right thigh’s medial side, featuring central calcification and multiple internal calcifications. The right deep femoral artery coursed within this mass, with a smaller lesion noted on the posterior thigh. Surgical consultation with a vascular surgeon led to planned intervention. The smaller mass was completely excised, but the larger one partially, as it encased the femoral artery. The inability to remove all HO was due to excessive bleeding. Postoperatively, the patient experienced no complications, and one-year follow-up revealed a favorable recovery with restoration of full right lower limb mobility.

**Conclusion:**

This case underscores the potential gravity of vascular injury associated with heterotopic ossification. Surgeons should remain vigilant regarding the risk of vascular injury during HO excision.

## Background

Heterotopic ossification (HO) is a pathological condition characterized by abnormal bone formation in non-ossified tissues. This condition involves the gradual accumulation of calcified bone within soft tissues, leading to symptoms such as joint swelling, pain, dysfunction, and the potential for joint arthrodesis [1]. Notably, instances of arterial entrapment or injury due to HO following long bone fractures are exceedingly rare [2, 3]. The irritation or damage of an artery within the fracture, whether occurring at the time of injury, during reduction, or during the subsequent formation of bony callus, can pose severe risks, including potentially life-threatening consequences [4–7].

In this case report, we present a unique case involving a 56-year-old male who experienced femoral artery injury and the organization of hematoma a decade after undergoing internal fixation with an intramedullary nail following a femoral shaft fracture. These complications stemmed from the development of HO.

## Case presentation

A 56-year-old male with a body mass index of 26.8 kg/m² presented at our clinic. Ten years prior, the patient had sustained bilateral femoral shaft fractures and a mild head injury following a motor vehicle accident. His initial treatment involved intramedullary nailing for the right femoral shaft fracture and the use of an internal fixation plate for the left femoral shaft fracture at a local hospital. Postoperatively, he exhibited excellent healing progress. Three months after the surgery, he was ambulatory with normal lower limb function. However, the patient noticed the gradual appearance of a hard mass in his right medial thigh, for which he did not seek immediate medical attention.

Five months prior to presentation, the patient experienced severe swelling and pain in his right thigh due to idiopathic muscle spasms, prompting a visit to a local hospital. A vascular ultrasound examination conducted at that time revealed femoral artery injury and the formation of a hematoma. The patient received a compression bandage, which temporarily alleviated the symptoms. Nevertheless, the thigh swelling persisted and solidified into a palpable mass.

Three months prior to presentation, the patient developed swelling and pain in his right thigh with no apparent cause. Compression bandage was once again applied at the local clinic, but the swelling progressively solidified, impairing his ability to walk.

Upon physical examination, we observed pronounced swelling and darkened skin in the medial and posterior regions of the right thigh, with the presence of two firm masses of varying sizes. These masses exhibited limited mobility, lacked tenderness, and featured well-defined boundaries. The local skin temperature was notably reduced, and there was no superficial vascular swelling or vascular murmurs. Notably, arterial pulses in the tibialis posterior, dorsalis pedis, and popliteal arteries were palpable. The ankle-brachial index (ABI) measured ≥ 0.9.

**Fig. 1:**
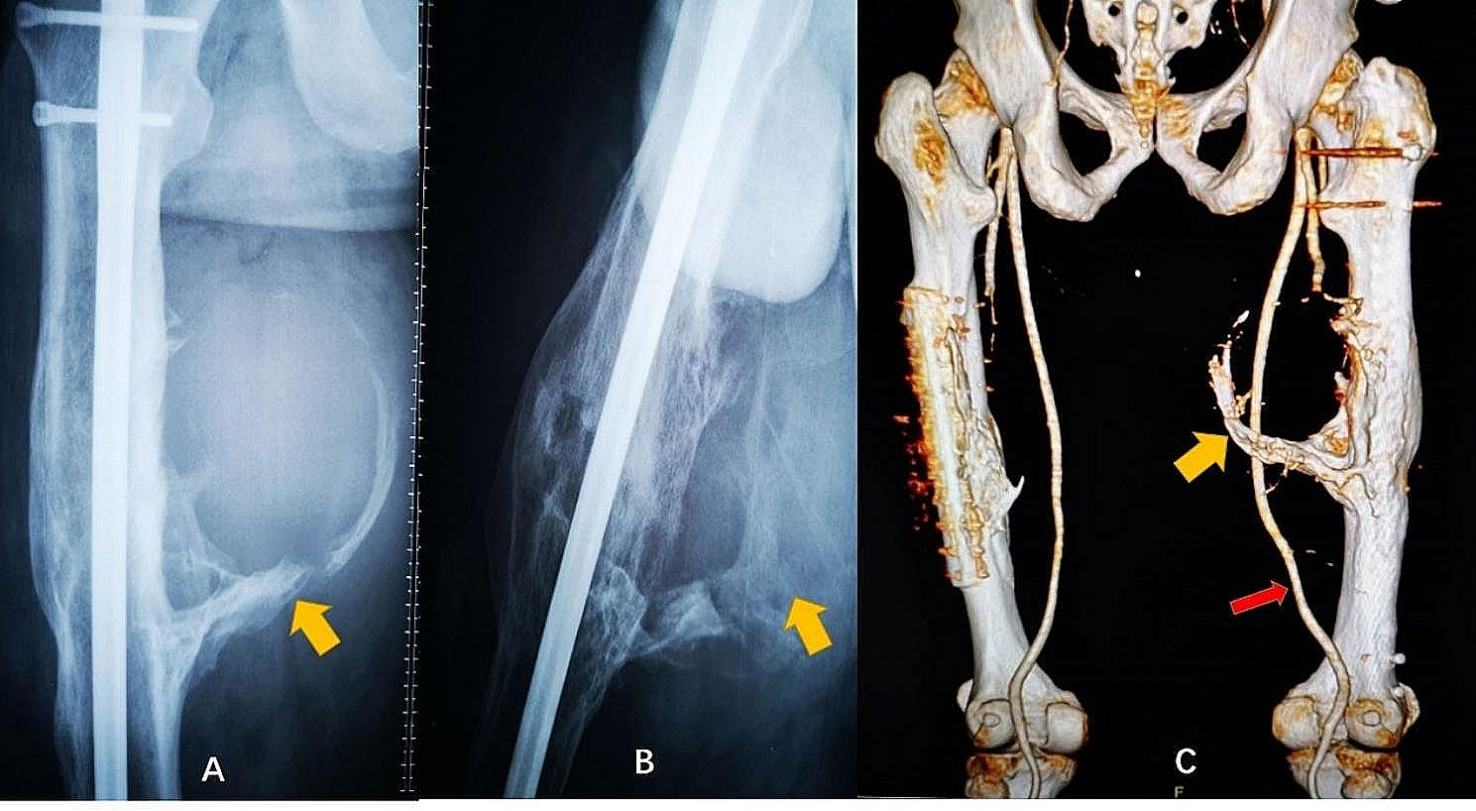
Preoperative radiographs. Anteroposterior (**A**), lateral radiographs (**B**), and three-dimensional CTA images (**C**) demonstrating severe heterotopic ossification (yellow arrow) in the medial-posterior part of the right thigh. The femoral profound artery (red arrow) is seen nearby the heterotopic ossification

Radiographs of both femoral shafts revealed postoperative changes, with satisfactory fracture healing and a prominent, high-density shadow along the inner and posterior aspects of the right thigh (Fig. 1A, B). Computed tomography angiography (CTA) disclosed a large mass with mixed density on the inner side of the right thigh, measuring 16.8 × 14.8 × 20.7 cm. This mass exhibited central calcification and multiple internal calcifications, with the right deep femoral artery traversing it (Fig. 1C). An additional smaller mass was identified on the lateral side of the posterior thigh.

Ultrasonographic findings indicated no substantial varicose vein migration through the great saphenous vein or the small saphenous vein on the right. The cluster in the mid-back of the right thigh, along with two adjacent low-echo, light clusters, measured approximately 20.35 cm in diameter. These displayed fluid echoes upon compression, suggestive of arteriosclerosis affecting the right lower extremity, potentially arising from the hypoechoic closure of the upper front and back of the right thigh. The hematoma had undergone organization, with both echoes exhibiting very low echogenicity.

Following consultation with a vascular surgeon, we considered heterotopic ossification to be the likely cause of the femoral artery injury’s recurrence. Consequently, the patient was scheduled for a planned surgical intervention.

**Fig. 2:**
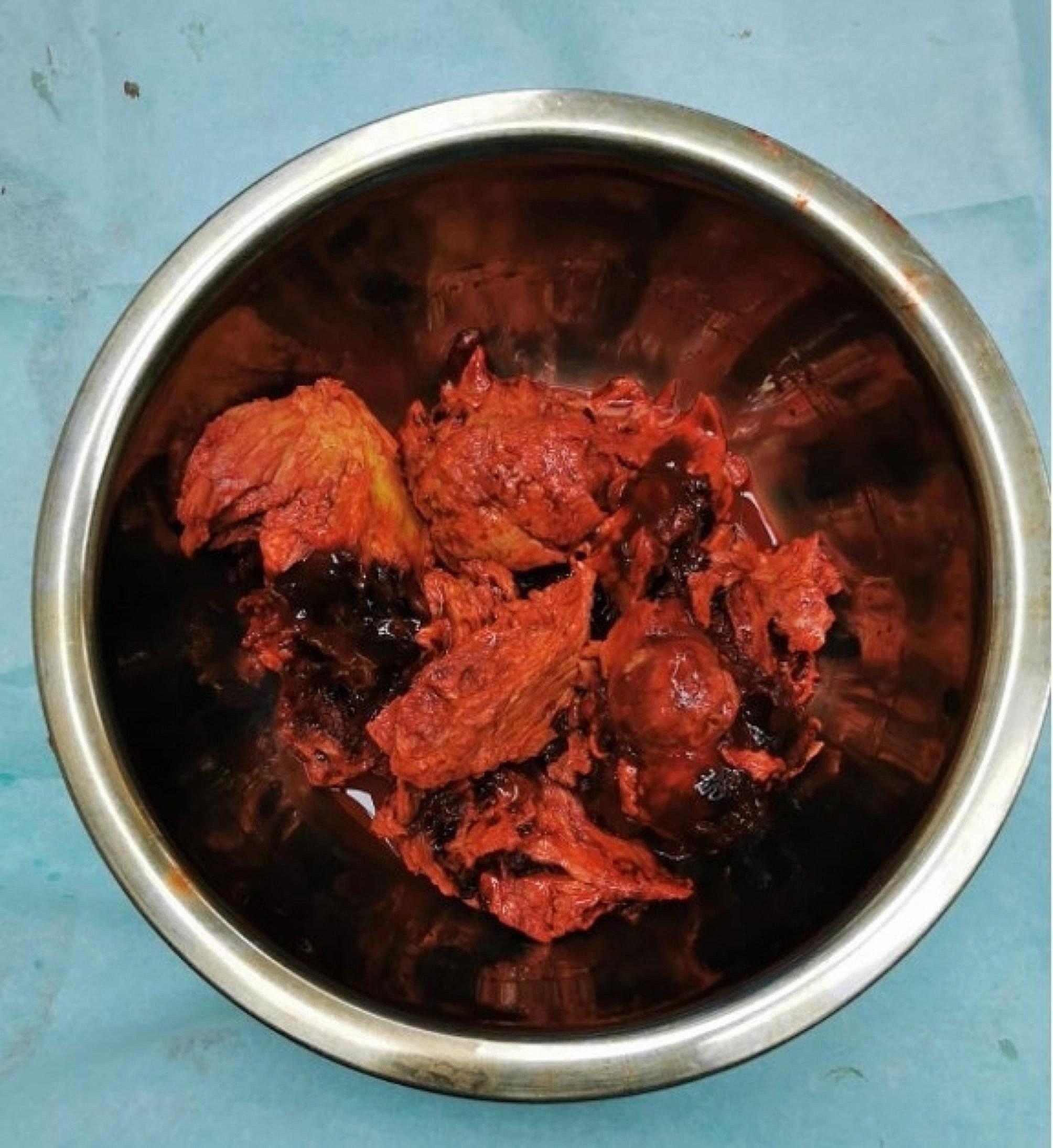
Intraoperative picture of the removed mass

Surgery and Pathology: Under general anesthesia, the patient was placed in a prone position. A longitudinal incision was made between the two masses, exposing a 20 cm × 15 cm × 15 cm mass on the medial thigh and an 8 cm × 6 cm × 6 cm mass on the lateral thigh. These masses were situated on the deep surface of the biceps femoris, with several nourishing blood vessels entering them. The posterior medial mass encased the callus and the femoral artery within a complete capsule. The smaller mass was excised, while the larger mass, due to its encasement of the femoral artery, could only be partially removed. The excised specimen was a solid mass (Fig. 2).

Intraoperative bleeding was profuse, and identifying a specific large bleeding point proved challenging, even with the assistance of a vascular surgery specialist. Despite attempts, not all heterotopic ossification could be removed due to the excessive bleeding. A blood transfusion of 2000 mL was administered.

**Fig. 3:**
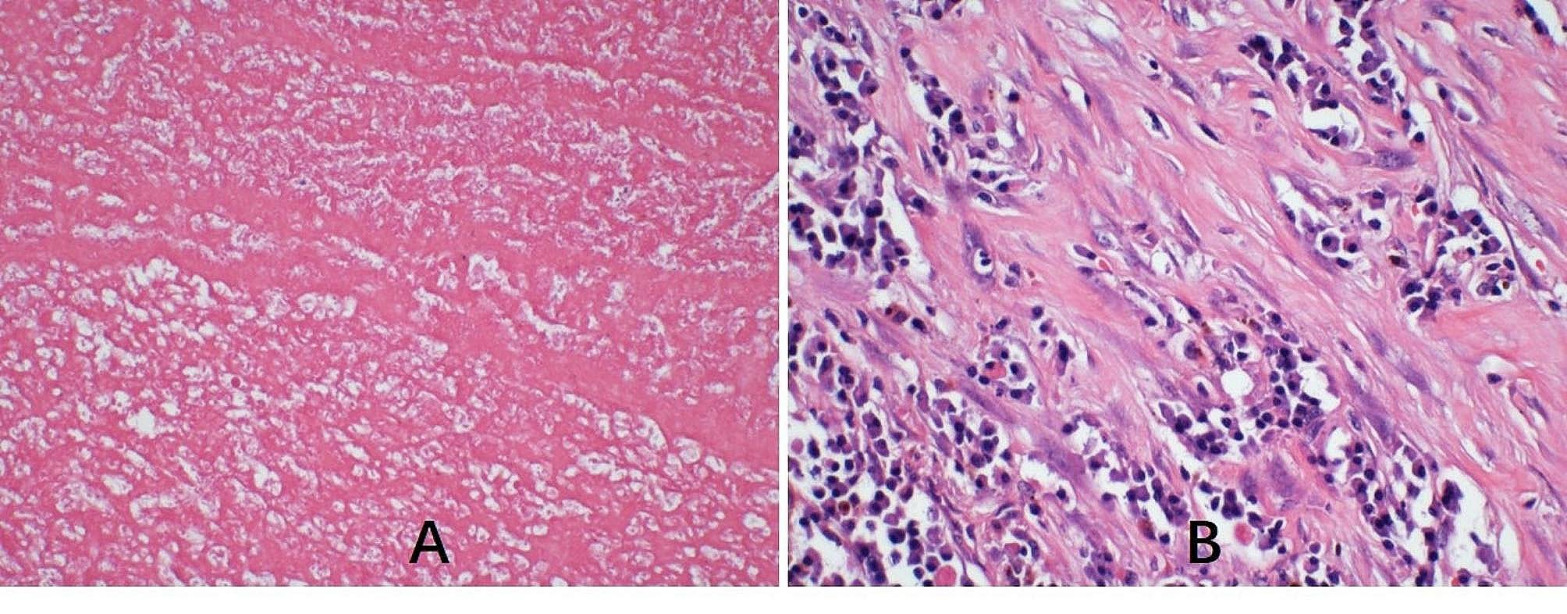
Pathological examination showing organization of old hematoma in the removed mass (**A**: H&E x100, **B**: H&E x200)

Pathological examination revealed a mass with a fragile texture, smooth surface, the presence of a capsule, extensive old clots, necrotic tissues, a lack of cellular and tissue structure, and degeneration of the fibrous capsule with granulation tissue on the exterior. The mass was diagnosed as an organized old hematoma (Fig. 3).

**Fig. 4:**
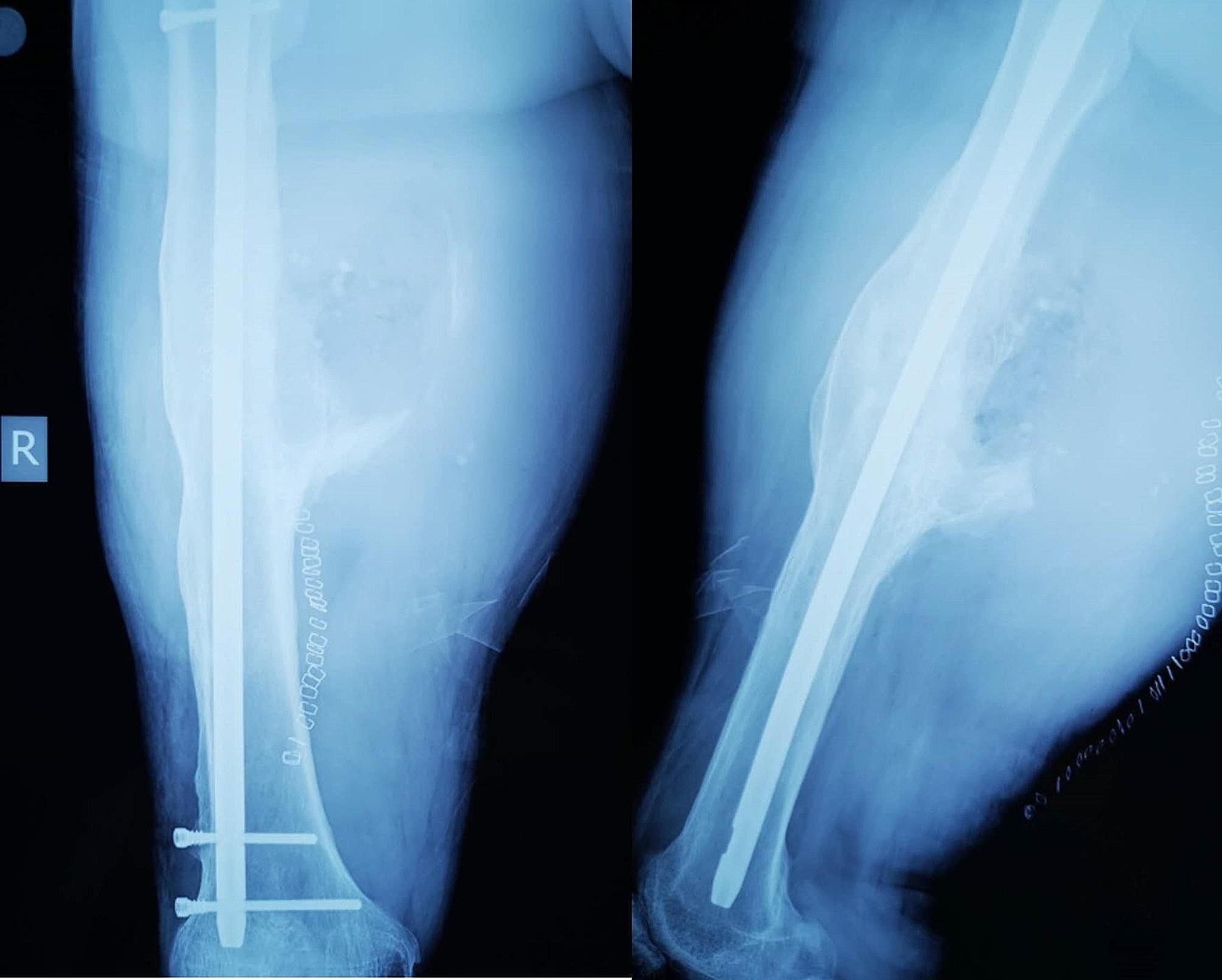
Postoperative radiographs showing partial removal of the heterotopic ossification

Postoperatively, the affected limb was treated with a compression bandage, and intravenous tranexamic acid was administered for five days. The patient was prescribed indomethacin 25 mg three times daily for eight weeks. Postoperative radiographs indicated partial removal of heterotopic ossification (Fig. 4). The patient resumed weight-bearing walking one-week post-surgery, and despite the absence of a strict rehabilitation regimen, he achieved a robust recovery, regaining full mobility of the right lower limb. Throughout the postoperative follow-up, no vascular injuries or associated complications were observed in the right thigh, and further development of heterotopic ossification was halted. The patient expressed satisfaction with the treatment and recovery process.

## Discussion and conclusion

HO is a recognized complication following fractures, and its incidence is influenced by a combination of external and internal factors. Reported rates of HO occurrence in the literature vary widely, ranging from 9–90% [8, 9]. Typically, HO develops between 4 and 24 weeks post-injury, with a peak incidence at around 8 weeks. However, in some cases, it can emerge several years after the initial injury. The majority of HO cases are asymptomatic, but a minority may result in complications such as complex regional pain syndrome, joint stiffness, nerve entrapment, osteoporosis, and pressure ulcers [8, 9]. While existing research primarily focuses on the involvement of nerves in HO, studies pertaining to vascular complications are limited. Vascular entrapment due to HO has been scarcely documented, with only a few reports by Whitely and Dixon [2], and Polfer [3]. Whitely and Dixon reported an instance of an axillary artery encased within the callus of a four-part fracture-dislocation of the proximal humerus in a 63-year-old woman [2]. Polfer et al. [3] detailed two cases of heterotopic ossification causing vascular entrapment in the lower extremity, resulting from blast trauma. Notably, these four cases did not exhibit signs of vascular injury. In these four cases, there were no signs of vascular injury. In the present case, there was no evidence of callus formation around the blood vessel. Instead, organizing hematoma was found surrounding the vessel, highlighting a unique clinical scenario. To the best of our knowledge, this case represents the first documented instance of symptomatic vascular injury associated with the organization of hematoma due to hypertrophic HO following a femoral shaft fracture.

HO is a pathological bone formation. According to the related factors of HO formation, heterotopic ossification can be divided into four types [9]: post-traumatic heterotopic ossification, neurogenic heterotopic ossification, and hereditary heterotopic ossification and other rare causes of heterotopic ossification. Heterotopic ossification after trauma is the most common [10]. The study found that the probability of traumatic brain injury and HO are positively correlated. The patient in this case also had a mild traumatic brain injury. Researchers believe that brain injury promotes the development of heterotopic ossification because: (1) There are target cells that can produce heterotopic ossification. (2) There are osteogenic factors that cause heterotopic ossification. (3) The microenvironment causing heterotopic ossification continues to exist [11, 12]. When brain injury is combined with a fracture, surgery is often performed on the fracture, which is also a risk factor for increasing the incidence of heterotopic ossification [13]. This is because, during surgical treatment, internal fixation is commonly used. This kind of fixation can compress the local area and make the blood supply of the microcirculation insufficient, leading to hypoxia and promoting heterotopic ossification.

What is noteworthy in this case is that the patient experienced bilateral femoral shaft fractures. Despite the similarity of the fracture sites, significant heterotopic ossification developed around the intramedullary nail on the right side, while the left side, managed with open reduction and plate fixation, did not exhibit pronounced heterotopic ossification. This observation raises an intriguing question regarding whether long tubular bones are inherently more susceptible to heterotopic ossification when managed with intramedullary fixation, a subject that warrants further investigation.

In the early stages, drugs and radiotherapy are usually used to prevent heterotopic ossification from getting worse. For severe patients, who have affected joint mobility or have symptoms of compression, surgical treatment is required to remove the ectopic bone. Excision of heterotopic bone is associated with relatively high rates of major short-term complications including neurovascular injury, substantial blood loss, increased hospital stay, fracture, and infection [12, 14]. Our aim was not to remove all heterotopic bone if doing so would sacrifice a substantial amount of uninvolved soft tissue or cause massive bleeding. In the present case, we removed only the HO that was nearby the vascular structures.

The present case is a chronic injury, and the blood vessels have already had more proliferation and compensation. This may have also been the cause of massive bleeding during the operation (compensatory blood vessel damage). The blood vessel was too close to the callus, and the corresponding compensatory branching blood vessels were injured when the callus was removed. In addition, even if blood vessels are surrounded by callus, a vascular crisis may not occur. Komnenou investigated the behavior of the brachial artery enclosed in a HO, by experimentally producing fractures of the humerus in 22 dogs. They found that HO may engulf an artery without interfering with its patency and blood flow, unless complicated by an infection that causes vascular occlusion [15]. Nevertheless, physicians should still be aware of the extreme and catastrophic situation of vascular crisis caused by hyperplasia of callus and damages to blood vessels. In the case described by Whitely and Dixon [2], during the open reduction of the fracture, the artery that protruded from the callus was incised by accident. For HO that may damage blood vessels, if surgical treatment is taken, preoperative CTA examination can help surgeons better understand the spatial relationship between callus and main arteries, to avoid damage to blood vessels during surgery.

Most hematomas are organized in the abdominal cavity after trauma [16]. Hematomas in the extremities are rare, and are more common in patients with hemophilia [17]. The heterotopic ossification in this case was too large, and the hyperplastic callus formed a sharp dagger-like shape. This HO irritated the surrounding muscles, causing the muscles to spasm. Furthermore, the HO is close to the femoral artery, and the muscle spasm compresses the femoral artery, causing the femoral artery to be damaged by the sharp callus. The damaged blood vessel bleeds, causing blood to pool in the intermuscular space to form a hematoma, and increasing the pressure of the hematoma to cause hemostasis. Because of the repeated stimulation by the callus and chronic repeated bleeding, the hematoma gradually worsens, the inside becomes organized, and the outside forms a capsule.

We would like to make the following recommendations based on this case. Firstly, surgeons should pay attention to the possibility of vascular damage by HO. Secondly, in the process of joint loosening and resection of heterotopic ossification, it is necessary to be alert to the possible damage to blood vessels. Removing hematoma and callus may cause massive bleeding thus surgeons should be vigilant and take preventive measures.

In conclusion, we have presented a unique case involving femoral artery injury and hematoma formation as a consequence of heterotopic ossification following intramedullary fixation for a femoral shaft fracture. Given the potentially devastating implications of arterial injury, surgeons should maintain a heightened awareness of the potential for vascular damage when undertaking the excision of heterotopic ossification.

## Data Availability

The dataset supporting the conclusions of this article is included within the article.

## Abbreviations

HO: Heterotopic ossification
ABI: Ankle-brachial index
CTA: Computed tomography angiography
